# Association of Thyroid Function and Severity of Illness in Liver Cirrhosis as Measured by Child-Pugh Score

**DOI:** 10.7759/cureus.36618

**Published:** 2023-03-24

**Authors:** Aswin Raj, Gopalakrishna Pillai, Arun Divakar, Vishnu Shivam, Anjaly Nair

**Affiliations:** 1 Internal Medicine, Amrita Institute of Medical Sciences and Research Centre, Kochi, IND; 2 Department of General Medicine, Coimbatore Medical College and Hospital, Coimbatore, IND; 3 Biostatistics, Amrita Institute of Medical Sciences and Research Centre, Kochi, IND

**Keywords:** thyroid metabolism and liver cirrhosis, thyroid hormones, tft: thyroid function test, thyroxine (t4), triiodothyronine (t3), thyroid stimulating hormone tsh, child-pugh (c-p) classification, live cirrhosis

## Abstract

Objective

The main aim of this study is to understand the existing knowledge gap between thyroid function tests and the severity of liver cirrhosis as measured by the Child-Pugh score.

Materials and methods

This is a cross-sectional study conducted on 100 patients diagnosed with cirrhosis of liver. Serum triiodothyronine (free T3), thyroxine (free T4), and thyroid stimulating hormone (TSH) levels were measured, and the severity of liver cirrhosis was measured by Child-Pugh score and statistical analysis were done to investigate the association of free T3, free T4 and TSH levels with Child-A, Child-B, and Child-C severity groups.

Results

The results revealed that there is a statistically significant positive correlation between TSH levels and Child-Pugh score, whereas a statistically significant negative correlation was associated between free T3 (fT3), free T4 (fT4) levels, and Child-Pugh Score. Further, we also observed that the Child-C group has 7.5-fold risk of increased TSH levels (odds ratio {OR} = 7.553, 95% CI = 2.869-19.883, p = 0.000), has 5-fold risk of decreased fT3 levels (OR = 5.023, 95% CI = 1.369-18.431, p = 0.009) and has 6.4-fold risk of decreased fT4 levels (OR = 6.402, 95% CI = 2.516-16.290, p = 0.000).

Conclusion

Our results demonstrated that there is a positive and direct correlation associated between increasing TSH with severity of liver cirrhosis as measured by Child-Pugh score, whereas a negative and inverse correlation was observed between decreasing fT3 and fT4 levels with the severity of liver cirrhosis as measured by Child-Pugh score. This suggests that the Child-Pugh score can be used as a prognostic indicator in cirrhotic patients.

## Introduction

Liver cirrhosis is one of the primary causes of morbidity and mortality worldwide with its prevalence increased by 74.53% from 1990 to 2017 [1,2]. It is the 11^th^ leading cause of mortality and 15^th^ leading cause of morbidity, accounting for 1.32 million deaths in 2017 [1]. It is classified clinically as "compensated" or "decompensated". Ascites is the most typical initial sign [3]. The preventable causes are alcohol consumption, viral hepatitis, and non-alcoholic fatty liver disease. To explain, the thyroid hormones maintain metabolic and thermogenic homeostasis and act by binding to thyroid hormone receptors, which are essential for cell differentiation throughout development [4]. As liver is the most crucial organ in the peripheral conversion of tetraiodothyronine (T4) to tri-iodothyronine (T3) by type 1 deiodinase enzyme, it is essential for thyroid hormone metabolism [5].

Type I deiodinase enzyme, the most common enzyme for thyroid metabolism in liver, is in charge of producing 30% to 40% of extrathyroidal T3. It converts T4 to T3 via 5' and 5 deiodination. In cirrhotic individuals, higher levels of TSH, low free T3 levels, and increased reverse T3 levels are the most typical thyroid hormone abnormalities. As bound T4 converts into T3, free T4 level is the first to show these abnormalities and is often important to measure clinically. Decreased activity of type 1 deiodinase enzyme decreases the conversion of T4 to T3 and is most likely to cause these abnormalities [6]. Consequently, hypothyroidism and low T3 syndrome are frequently described in patients with liver cirrhosis. With this, T3 and T4 levels are reduced due to inadequate hepatic deiodination and poor hepatic cellular absorption. The most likely cause of the reduction in T4 levels is either the activity of peripheral-binding inhibitors or a lack of thyroid-binding globulin (TBG) synthesis [7]. An increase in T4 and TBG, as well as an increase in acute phase proteins, can be caused by acute liver disease and primary biliary cirrhosis. A meta-analysis reported that decreased free T3 (fT3) and free T4 (fT4) levels were significantly associated with a higher risk of liver cirrhosis, whereas increasing TSH levels were positively associated with the risk of liver cirrhosis [8]. Hence, our study aims to understand further the existing knowledge gap concerning the association between thyroid function test (fT3, fT4 and TSH levels) and the severity of liver cirrhosis by using the Child-Pugh score.

## Materials and methods

This is a cross-sectional study conducted at Amrita Insitute of Medical Sciences, Kerala, India, after obtaining ethical clearance from the Ethics committee of Amrita Institue of Medical Sciences with the approval number ECASM-AIMS-2022-003. A minimum sample size of 20 subjects was calculated based on a small pilot study conducted on 10 subjects with 80% power and 95% confidence and our study was conducted with 100 subjects. Subjects included in our study were diagnosed with cirrhosis of liver by clinical, biochemical, and radiological evidence of cirrhosis. Child-Pugh score was used for assessing the severity of liver cirrhosis. Patients with sepsis, pregnancy, cardiac failure, personal history of thyroid disorder, and drugs that alter thyroid functions were excluded from our study. The main objective of our study is to investigate the association between thyroid function test (Free T3, free T4, and TSH levels) and the severity of liver cirrhosis as measured by the Child-Pugh score. All enrolled patients were thoroughly evaluated by detailed history and clinical examination. Investigations such as complete blood count, renal function test, liver function test, prothrombin time, international normalized ratio (INR), and antibodies against HIV, Hepatitis C, and Hepatitis B were carried out. Ultrasound abdomen and fasting thyroid profile including free T3 (fT3), free T4 (fT4), and TSH were investigated in all patients enrolled in our study.

Statistical analysis

Statistical analysis was performed using IBM SPSS version 20.0 software (IBM Corp., Armonk, NY). Pearson Chi-square test was used as a statistical test of significance and a p-value of less than 0.05 was considered statistically significant. Odd ratios were calculated with 80% power and 95% confidence intervals to estimate the associations of fT3, fT4, and TSH levels with the severity of liver cirrhosis measured by the Child-Pugh score.

## Results

**Figure 1:**
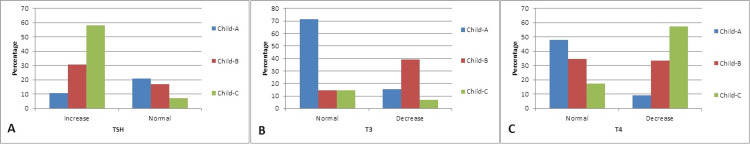
Percentage distribution of TSH (A) T3 level, (B) T4 level, and (C) liver cirrhosis as measured by the Child-Pugh score. TSH, Thyroid Stimulating Hormone; T3, Tri-iodothyronine; T4, Thyroxine.

Out of the 100 patients included in the study, 66 patients were males (66%) and 34 were females (34%) aged between 30 and 80 years. The etiology of cirrhosis was found to be alcohol (47%), viral (20%), nonalcoholic liver disease (29%) and cryptogenic (4%). Our results as shown in Table 1 and depicted in Figure 1 shows that 55 patients (55%) had increased TSH levels and 45 patients (45%) had normal TSH levels; 79 patients (79 %) had decreased free T3 (fT3) levels and 21 patients (21%) had normal fT3 levels; 54 patients (54%) had decreased free T4 (fT4) levels and 46 patients (46%) had normal fT4 levels. Correlation analysis of TSH and Child-Pugh score (r = 0.404) shows that there is a statistically significant positive correlation between TSH and Child-Pugh score with a p-value < 0.001. The correlation analysis of fT3, fT4 levels, and Child-Pugh score shows that there is a statistically significant negative correlation between fT3 (r = -0.404), fT4 (r = -0.528) and Child-Pugh Score with p-value < 0.001.

**Table 1:** Statistical analysis showing the association of TSH, T3, T4, and cirrhosis of liver as measured by the Child-Pugh score TSH, Thyroid Stimulating Hormone; T3, Tri-iodothyronine; T4, Thyroxine. Child-Pugh Score: Child- A, Child-B, and Child-C. Pearson Chi-square test was used as a statistical test of significance, and p < 0.05 was considered statistically significant.

TSH	Increased n=55	Normal n=45	p-value	Odds ratio (95% Confidence Interval)
Child – A n(%)	6(10.9%)	21(46.7%)	0.000	0.139 (0.050-0.392)
Child – B & C n(%)	49(89.1%)	24(53.4%)		
Child – B n(%)	17(30.9%)	17(37.8%)	0.235	0.736 (0.321-1.691)
Child – A & C n(%)	38(69.1%)	28(62.3%)		
Child – C n(%)	32(58.2%)	7(15.6%)	0.000	7.553 (2.869-19.883)
Child – A & B n(%)	23(41.8%)	38(84.4%)		
T3	Decreased n=79	Normal n=21	p-value	Odds ratio (95% Confidence Interval)
Child – A n(%)	12(15.2%)	15(71.4%)	0.000	0.071 (0.023-0.221)
Child – B & C n(%)	67(84.8%)	6(28.6%)		
Child – B n(%)	31(39.2%)	3(14.3%)	0.020	3.8750 (1.053-14.261)
Child – A & C n(%)	48(60.8%)	18(85.7%)		
Child – C n(%)	36(45.6%)	3(14.3%)	0.009	5.023 (1.369-18.431)
Child – A & B n(%)	43(54.4%)	18(85.7%)		
T4	Decreased n=54	Normal n=46	p-value	Odds ratio (95% Confidence Interval)
Child – A n(%)	5(9.3%)	22(47.8%)	0.000	0.111 (0.038-0.330)
Child – B & C n(%)	49(90.7%)	24(52.2%)		
Child – B n(%)	18(33.3%)	16(34.8%)	0.439	0.9375 (0.409-2.147)
Child – A & C n(%)	36(66.7%)	30(65.2%)		
Child – C n(%)	31(57.4%)	8(17.4%)	0.000	6.402 (2.516-16.290)
Child – A & B n(%)	23(42.6%)	38(82.6%)		

Further subgroup analysis for odds ratio (OR) shows that increased levels of TSH, decreased levels of fT3 and fT4 are significantly associated with increased severity of liver cirrhosis as measured by Child-Pugh score as shown in Table 1. 10.9% of Child-A (n=6), 30.9% of Child-B (n=17) and 58.2% of Child-C (n=32) had increased TSH levels and 46.7% of Child-A (n=21), 37.8% of Child-B (n=17), 15.6% of Child-C (n=7) had normal TSH levels as shown in Table 1. 15.2% of Child-A (n=12), 39.2% of Child-B (n=31) and 45.6% of Child-C (n=36) had decreased T3 levels and 71.4% of Child-A (n=15), 14.3% of Child-B (n=3) and 14.3% of Child-C (n=3) had normal T3 levels as shown in Table 1. 9.3% of Child-A (n=5), 33.3% of Child-B (n=18), 57.4% of Child-C (n=31) had decreased T4 levels and 47.8% of Child-A (n=22), 34.8% of Child-B (n=16) and 17.4% of Child-C (n=8) had normal T4 levels as shown in Table 1. Further, we also observed that the Child-C group has 7.5-fold risk of increased TSH levels with OR = 7.553 (95% CI {confidence interval} = 2.869-19.883, p = 0.000), has 5-fold risk of decreased fT3 levels with OR = 5.023 (95% CI = 1.369-18.431, p = 0.009) and has 6.4-fold risk of decreased fT4 levels with OR = 6.402 (95% CI = 2.516-16.290, p = 0.000).

## Discussion

Our results revealed that thyroid dysfunction of increased TSH levels, decreased fT3 levels and fT4 levels were significantly associated with the severity of patients with liver cirrhosis as measured by the Child-Pugh score. A Low T3 syndrome may also be accompanied by chronic liver illness. According to our study, alcohol is the leading contributor to cirrhosis, followed by non-alcoholic liver disease which is consistent with the previous study [9]. In our study out of 100 patients, 55 patients had elevated TSH levels and 45 patients had normal TSH levels as shown in Table 1. Among these, 10.9% of patients in the Child-A group had increased TSH levels, 30.9% of patients in the Child-B group had increased TSH Levels and 58.2% of patients in the Child-C group had increased TSH levels, which is consistent with the results reported by previous studies [10-12]. This revealed that there is a statistically significant 7.5-fold risk of increased TSH levels with Child-C group of severity in patients with liver cirrhosis as measured by Child-Pugh score and is shown in Table 1. A recent study [13] reported the existence of aberrant TSH levels in liver cirrhosis which is consistent with our results. Thus, our results showed that there is a significant positive correlation between the TSH levels and the severity of liver cirrhosis as measured by the Child-Pugh Score [14]. The results also revealed that 55% of patients with cirrhosis had hypothyroidism with high TSH levels, decreased fT3 levels and variable fT4 levels. This indicates that the prevalence of hypothyroidism increases as the severity of liver cirrhosis increases. The TSH levels of all 55 patients were also within the subclinical range (TSH = 7-10 mIU/L) of hypothyroidism despite the absence of any clinical symptoms of hypothyroidism.

Our results showed that fT3 levels were found to be decreased in 79% of cirrhotic patients as shown in Table 1. Among this 44% of patients in the Child-A group had decreased T3 levels, 91% of patients in the Child-B group had decreased T3 Levels and 92% of patients in the Child-C group had decreased T3 levels. This demonstrates that there is a significant 3.8-fold risk and 5-fold risk of decreased T3 levels with Child-B and Child-C groups of severity in patients with liver cirrhosis as measured by Child-Pugh score as shown in Table 1. Thus, fT3 levels were inversely correlated with the Child-Pugh score as demonstrated in our study and are consistent with the results shown by the previous study [4,15].

Our results showed that 54% of patients had decreased fT4 levels and 46% of patients had normal fT4 levels as shown in Table 1. Among this 9.3% of patients in the Child-A group had decreased fT4 levels, 33.3% of patients in the Child-B group had decreased fT4 Levels and 57.4% of patients in the Child-C group had decreased fT4 levels. This demonstrates that there is a statistically significant 6.4-fold risk of decreased fT4 levels with the Child-C group of severity in patients with liver cirrhosis as measured by Child-Pugh score as shown in Table 1. Thus, fT4 levels show a significant negative correlation with the severity of liver dysfunction as measured by the Child-Pugh score, which is consistent with the results reported by the previous study [4,13].

The most prevalent thyroid function abnormality associated with liver cirrhosis is hypothyroidism and the second most frequent thyroid function abnormality associated with liver cirrhosis is low T3 syndrome. Abnormal thyroid function was prevalent in patients with liver cirrhosis. The possible explanation could be that, since the liver is the primary site for the conversion of T4 to T3, a decrease in T3 conversion indicates that the severity of liver disease directly affects the deiodination process of thyroid hormones rather than having an indirect impact on systemic illness.

Limitations

The main limitation of our study is the small sample size and lack of detailed subgroup-level analysis of age, gender, and etiology with liver cirrhosis severity as measured by the Child-Pugh score and thyroid function test. Further, multi-center prospective studies with large sample sizes are needed to validate and generalize these results for clinical implementation.

## Conclusions

In conclusion, our results demonstrated that abnormal thyroid function tests were significantly associated with the severity of liver cirrhosis as measured by the Child-Pugh score. Our study revealed that there is a statistically significant association between the severity of liver cirrhosis as measured by Child-Pugh score with increased TSH levels, decreased fT3 and fT4 levels. Further, results showed that there is a positive and direct correlation associated between increasing TSH with the severity of liver cirrhosis as measured by Child-Pugh score, whereas a negative and inverse correlation was observed between decreasing fT3 and fT4 levels with the severity of liver cirrhosis as measured by Child-Pugh score. This suggests that the Child-Pugh score can be used as a prognostic indicator in cirrhotic patients, and increased TSH levels may indirectly help in identifying patients with poor prognosis. Moreover, future studies were needed to address the association of the severity of thyroid function tests with the grading of liver disease by Child-Pugh score.
